# Analysis of Bubble Growth in Supercritical CO_2_ Extrusion Foaming Polyethylene Terephthalate Process Based on Dynamic Flow Simulation

**DOI:** 10.3390/polym13162799

**Published:** 2021-08-20

**Authors:** Shun Yao, Yichong Chen, Yijie Ling, Dongdong Hu, Zhenhao Xi, Ling Zhao

**Affiliations:** 1 Shanghai Key Laboratory of Multiphase Materials Chemical Engineering, East China University of Science and Technology, Shanghai 200237, China; yaoshun0727@163.com (S.Y.); cccyccxc@163.com (Y.C.); lingyj000@163.com (Y.L.); hudd@ecust.edu.cn (D.H.); zhhxi@ecust.edu.cn (Z.X.); 2 School of Chemical Engineering and Technology, Xinjiang University, Urumqi 830046, China

**Keywords:** supercritical CO_2_, PET extrusion foaming process, bubble growth, dynamic melt flow

## Abstract

Bubble growth in the polymer extrusion foaming process occurs under a dynamic melt flow. For non-Newtonian fluids, this work successfully coupled the dynamic melt flow simulation with the bubble growth model to realize bubble growth predictions in an extrusion flow. The initial thermophysical properties and dynamic rheological property distribution at the cross section of the die exit were calculated based on the finite element method. It was found that dynamic rheological properties provided a necessary solution for predicting bubble growth during the supercritical CO_2_ polyethylene terephthalate (PET) extrusion foaming process. The introduction of initial melt stress could effectively inhibit the rapid growth of bubbles and reduce the stable size of bubbles. However, the initial melt stress was ignored in previous work involving bubble growth predictions because it was not available. The simulation results based on the above theoretical model were consistent with the evolution trends of cell morphology and agreed well with the actual experimental results.

## 1. Introduction

Recently, the theoretical understanding and quantitative prediction of the foaming process in dynamic polymer fluids have been of great significance for a wide range of industrial applications [1,2,3,4,5]. Typically, dynamic polymer foaming processes consist of a melt flow and diffusion-induced growth of gas bubbles in polymeric fluids under extrusion as well as injection molding conditions. Compared to static polymer foaming, polymer foaming under a dynamic shear and elongation field is full of high complexity, especially for the extrusion foaming process. Extrusion foaming processes usually consist of six stages: (1) the plasticized flow of polymer melts in extruder; (2) the dissolution and homogenization of the blowing agent in the polymer melt; (3) the cooling optimization process of the polymer/blowing agent solution by lowering the temperature to a suitable foaming temperature; (4) the shear and elongation flow of the polymer/gas homogenized fluid inside the die channel; (5) the diffusion-induced growth of bubbles in the molten polymer; (6) the stabilization and maturation of the cell structure by lowering the temperature below the glass transition temperature of the polymer [6,7,8,9,10].

Many efforts have been made to clarify the polymer foaming process at the aspects of either in situ experimental observations [11,12,13,14] or theoretical establishments [5,15,16]. The bubble growth is a complicated process that couples a mass-transfer process and momentum-transfer process. In situ visualization data intuitively show continuous changes of bubble growth during the polymer foaming process. These results directly reflect the effect of thermodynamic and rheological properties of the polymer/gas mixture on bubble growth, such as the surface tension, gas solubility, gas diffusivity, depressurization rate, zero-shear viscosity and relaxation time, etc. Meanwhile, the important results provided much valuable information to develop a theoretical model and numerical simulation scheme to describe the bubble growth process. Amon and Denson [15] proposed a mathematical analysis in an expanding foam based on a cell model. The well-known cell model described an important qualitative feature of a real system of a large number of cells growing in close proximity to one another. It divided the foam into numerous microscopic unit cells more realistically, which achieved a representative breakthrough in the field of bubble growth simulation. Arefmanesh [16] further developed the bubble growth model surrounded by a thin shell of viscoelastic fluid. The upper-convective Maxwell equation is employed to characterize fluid rheology behaviors. This provides a numerical solution for the bubble growth model of viscoelastic fluids. Shafi [17] presented a promising model to clarify the bubble nucleation and bubble growth behaviors simultaneously to investigate the effects of various situations on cell size distribution. Although some assumptions could not be in line with the actual foaming processes, they provided insights for more in-depth bubble growth simulation research. Taki [5] studied the effect of the pressure release rate on foaming, which is based on the kinetic experimental data about nucleation of bubbles under a finite depressurization rate, and the calculation results of cell density and bubble growth rate agreed well with experimental results. Li and Chen [1,2] adopted a bubble growth stability judgement to study the influence of various factors on bubble growth, so as to judge the stability time of bubble growth.

However, there are few studies on the bubble growth of dynamic polymer fluids. The rheological properties of polymer fluids in the dynamic extrusion process are difficult to determine and characterize through experiments, which resulted in that the analysis of bubble growth during the extrusion foaming process remains challenging. Therefore, it is necessary to simulate the polymer flowing process and solve the typical “black box” problem. Computational fluid dynamics (CFD) calculation is a wonderful solution to provide insight into the flow pattern [18,19,20,21,22]. Luo and Tanner [23,24] first introduced a finite streamline element method to solve viscoelastic flow simulation with K-BKZ integral constitutive equations. Numerical simulations realized a well agreement with experimental results for all situations with a shear rate of up to 10 s^−1^. Since then, the CFD-assisted extrusion process simulation and die design have achieved a blowout development. A. Garcia [25] accomplished the optimization of the swelling behavior through an appropriate die design with the aid of a computer-based simulation. It was found that the extrusion swell was strongly related to the die contraction ratio and vertical inclination angle. This greatly reduced the design cost and shortened the experiment periods. Ganvir [26] adopted the Arbitrary Lagrangian Eulerian method-based finite element formulation to predict the extrudate swell of commercial grades of linear polyethylene and branched polyethylene. The Phan-Thien–Tanner (PTT) constitutive equations were used to model the rheology of different polymers, and the simulation for the extrudate swell profile was in good agreement with the experimentally measured swell profile. Tang and R. D’hooge [27] estimated the parameters of the generalized Newtonian constitutive model with Cross law and the multimode viscoelastic PTT constitutive model from the rheological data. It was found that the PTT model could better predict three-dimension swell behaviors for the isothermal flow through the slit die of polypropylene. This is due to the lack of a mathematical description of the melt memory effect in Cross law. In summary, the PTT model based on the finite element simulation could realize good numerical predictions for the flow of polymer viscoelastic fluids. However, the current numerical simulation of extrusion foaming still assumed that the polymer was a Newtonian fluid [6]. The numerical simulation based on a CFD analysis of extrusion foaming for viscoelastic non-Newtonian polymer fluids is still in its infancy.

In this paper, the main objective is to provide insight into the complicated numerical simulation of the supercritical CO_2_ (scCO_2_) extrusion foaming polymer process, selecting polyethylene terephthalate (PET) as a reference polymer, since extruded PET foams have been commercialized and widely applied in structure core lightweight materials of wind power blades. Symmetrical 2D differential viscoelastic flow simulations were carried out with the multi-mode PTT model to calculate the flow pattern inside the die. The shear rate field, pressure profiles and melt stress distributions were calculated within the corresponding 2D simulation configuration. Through the dynamic rheological profiles of the PET melt in the cross section of the die outlet, the rheological characteristic parameters under dynamic flow conditions were acquired. In addition, the thermodynamic parameters of the PET/CO_2_ mixture were calculated based on the melt states in the die outlet. Then, these rheological and thermodynamic paraments were substituted into the bubble growth model for a numerical simulation. The simulation results confirmed the evolution trends of cell morphology and agreed well with the actual experimental results, which reflected the rationality and accuracy of the coupling scheme.

## 2. Theory and Governing Equations

An incompressible polymeric fluid in an isothermal extrusion flow can be modelled by the mass and momentum conservation equations. The applied governing equations involving continuity and momentum equations can be expressed as [27,28,29]:

(1) ∇·ν=0

(2) $$\rho \left(\frac{\partial \nu }{\partial t}+\nu \cdot \nabla \nu \right)=-\nabla p+\nabla \cdot \tau$$

where ν is the velocity, ρ is the density of the fluid, p is the pressure, τ is the stress tensor and is given by a constitutive equation.

### 2.1. The PTT Constitutive Equation

The PTT model is one of the most realistic differential viscoelastic models. The PTT model computes $T_{1i}$ from:

(3) $$\exp\left[\frac{\varepsilon_{i}\lambda_{i}}{\eta_{i}}tr\left(T_{1i}\right)\right]T_{1i}+\lambda_{i}\left[\left(1-\frac{\xi_{i}}{2}\right){\overset{ˇ}{T}}_{1i}+\frac{\xi_{i}}{2}{\hat{T}}_{1i}\right]=2\eta_{i}D$$

where ${\overset{ˇ}{T}}_{1i}$ and ${\hat{T}}_{1i}$ are the upper and lower convective derivatives of $T_{1i}$. Two non-linear parameters $\xi_{i}$ and $\varepsilon_{i}$ are in control of the shear and elongational behavior of the viscoelastic fluid.

$T_{2i}$ is computed from Equation (4):

(4) $$T_{2i}=2\eta_{2i}D$$

where D is the rate-of-deformation tensor and $\eta_{2i}$ is the viscosity factor for the purely viscous component of the extra-stress tensor. The viscosity ratio $r_{n}$ is defined as $\eta_{2i}/\eta_{1i}$. The relationship of $\eta_{1i}$ and $\eta_{2i}$ is expressed by:

(5) $$\eta_{1i}=\left(1-r_{n}\right)\cdot \eta_{i}$$

(6) $$\eta_{2i}=r_{n}\cdot \eta_{i}\;$$

When a multi-mode viscoelastic model is used, the purely viscous component of the extra-stress tensor is defined through the first mode only.

### 2.2. Bubble Growth Model

According to the bubble growth model, the diagram of a corresponding polymer/gas solution shell is shown in Figure S1. The following assumptions and principles were adopted in this work [1,2,5,15,16,17]:

(1) The bubble is spherically symmetric.
(2) The whole foaming process is isothermal.
(3) The PET/CO_2_ solution is incompressible.
(4) The gas concentration inside the bubble is related to the dissolved gas concentration at the interface obeying Henry’s law.
(5) The gravity can be neglected.
(6) The CO_2_ gas behavior follows the Peng–Robinson cubic equation of state.
(7) The effect of scCO_2_ plasticization is ignored.
(8) The bubble growth process does not start until PET/CO_2_ mixture leaves the die outlet.

On the basis of the above assumptions; the mathematical formulations are illustrated in SI Section S1.

### 2.3. Methods of Finite Element Calculations

A flow solver (Polyflow) was employed to calculate the flow conditions of the polymer melt in the die channel based on the governing equations and constitutive models. The finite element algorithm based on the discrete elastic viscous stress splitting (DEVSS) method and the streamline upwind (SU) scheme was adopted to promote the simulation stability and convergence process. In viscoelastic flows, the constitutive models are highly nonlinear. Therefore, the evolution procedure was applied in this work to greatly simplify the handling of the computational difficulties of nonlinear problems. The quadratic representation of the velocity field and a linear representation of the pressure field were used to guarantee calculation accuracy while sacrificing calculation costs.

**Figure 1:**
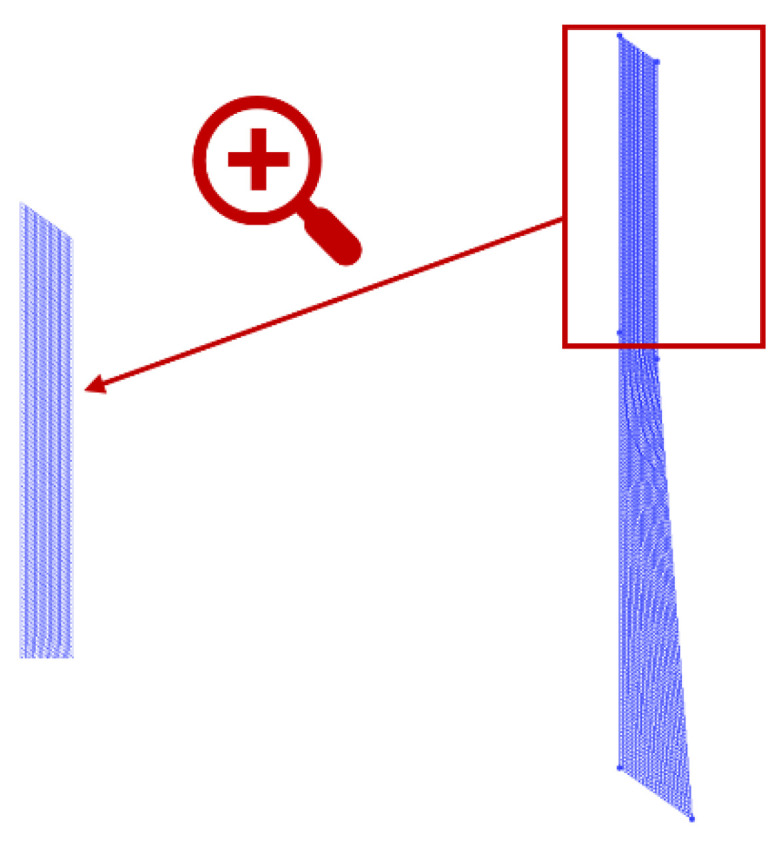
Schematic diagram of Mesh 2.

**Figure 2:**
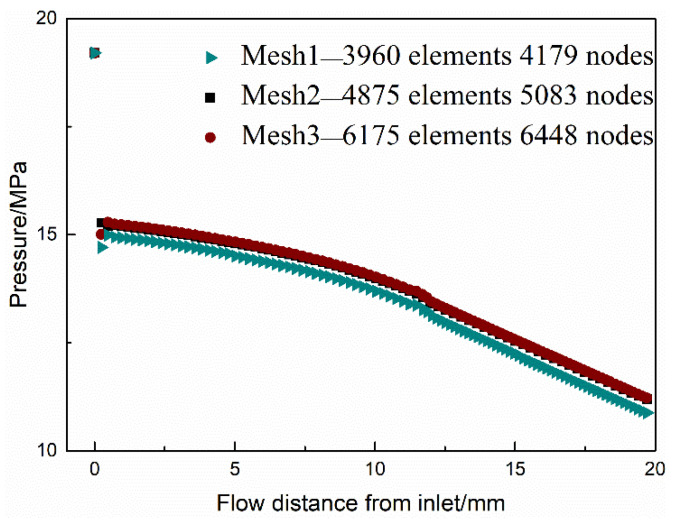
The simulated pressure profiles along the die wall for three mesh patterns.

Quadrilateral elements were accepted to implement the mesh within the ICEM package, which are shown in Figure 1. Along the die channel wall, the pressure profile predicted by the PTT model based on Mesh 1 was lower than those based on Mesh 2 and Mesh 3. It was shown in Figure 2 that the simulation results based on Mesh 2 and Mesh 3 were basically the same. This phenomenon illustrated that Mesh 2 and Mesh 3 had enough nodes to obtain more accurate pressure profiles compared with Mesh 1 [30]. Therefore, Mesh 2 with 4875 elements was determined to compromise in calculation accuracy and calculation costs in the subsequent study.

## 3. Experimental Section

### 3.1. Materials

High melt strength PET was purchased from Shanghai Petrochemical Co., Shanghai, China. The mass-average and number-average molar masses of the PET were 49,000 g/mol and 23,000 g/mol, respectively. CO_2_ (purity: 99.995% *w*/*w*) was purchased from Air Products Co., Shanghai, China. All the materials were used as received.

### 3.2. Rheology Characterization

To obtain the rheological data of PET melt at different foaming temperatures, the rheological properties were measured using a HAAKE Mars III (Thermo Fisher Scientific Inc. Co., Waltham, MA, USA) with a 35 mm disk under nitrogen. The frequency sweep was conducted in an angular velocity range of 0.005–300 rad/s.

### 3.3. PET Extrusion Foaming Process

**Figure 3:**
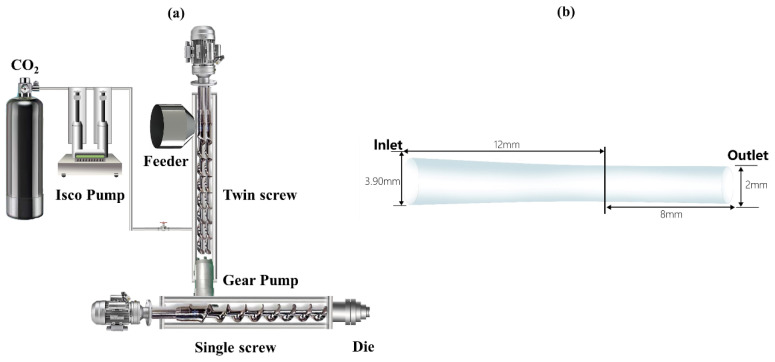
Schematic diagram of (**a**) extrusion foaming system; (**b**) the die channel.

A two-stage tandem extrusion foaming system was used to foam the PET samples. The tandem system consisted of a twin-screw and a single-screw. Figure 3a illustrated the schematic of the configuration of this extrusion foaming system. The first extruder was a twin-screw with L/D ratio of 44/1. The second extruder was a cooling single-screw with L/D ratio of 20/1. The PET/CO_2_ mixture foamed at the die exit, which was subjected to a rapid depressurization. The nucleation and bubble growth started owing to the thermodynamic supersaturation and mass transfer. Four PET foams with different foaming temperature (271 °C, 274 °C, 277 °C, 280 °C) were extruded and quickly cooled to solidify. These samples were named in accordance with the foaming temperature (e.g., PF represents PET foam, PF-271 represents that the foaming temperature was 271 °C).

### 3.4. Die Geometry and Boundary Conditions

The 2D flow domain for a given geometry is shown in Figure 3b. Due to the symmetry, only half of the die channel was established for all simulations in Polyflow to reduce the computational periods. The geometry parameters are also marked in Figure 3b.

A fully developed flow was assumed at the flow inlet. Moreover, a nonslip situation for the velocity field was prescribed. It indicated that the normal velocity and tangential velocity were equal to zero.

### 3.5. Foam Characterization

The cell morphologies of the PET foams were characterized by a scanning electron microscopy (SEM) (Nova NanoSEM450, FEI Ltd., Hillsboro, OR, USA). The samples were immersed in liquid nitrogen and then fractured. The SEM scanned fractured surface with Pd (palladium) coating. The average cell size was obtained through the analysis of the SEM photographs by the software of Image-Pro Plus. The number average diameter of all the cells in the micrograph, $d_{cell}$, was calculated using the following equation:

(7) $$d_{cell}=\frac{\sum d_{i}n_{i}}{\sum n_{i}}$$

where $n_{i}$ was the number of cells with a perimeter-equivalent diameter of $d_{i}$.

## 4. Results and Discussion

### 4.1. Rheological Parameter Tuning for PTT Model

**Figure 4:**
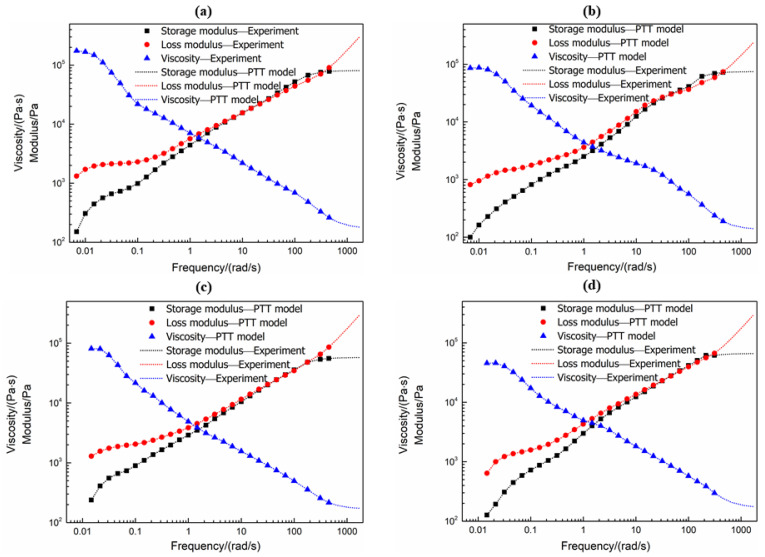
The experimentally recorder linear viscoelastic properties and PTT model fitting curves of PET melt: (**a**) 271 °C; (**b**) 274 °C; (**c**) 277 °C; (**d**) 280 °C.

The characterization of rheological properties with a small-amplitude oscillatory shear (SAOS) test was well established. Four-mode PTT models were obtained by fitting the master curves of linear viscoelastic data of the sample at four foaming temperatures of 271 °C, 274 °C, 277 °C and 280 °C, which can be seen in Figure 4. With the decrease in temperature, the relaxation activation energy of chain segments became larger to store more energy and the viscoelasticity effects became stronger. According to the Cox–Merz rule [31], the complex viscosity could agree well with the shear viscosity in the low shear rate range and high shear rate range [27]. Therefore, the establishment of PTT models successfully extended the rheological data to the high shear rate region. Table S1 includes the model parameters at four different temperatures by using the differential PTT models.

### 4.2. Rheological Properties under Dynamic Extrusion Conditions

**Figure 5:**
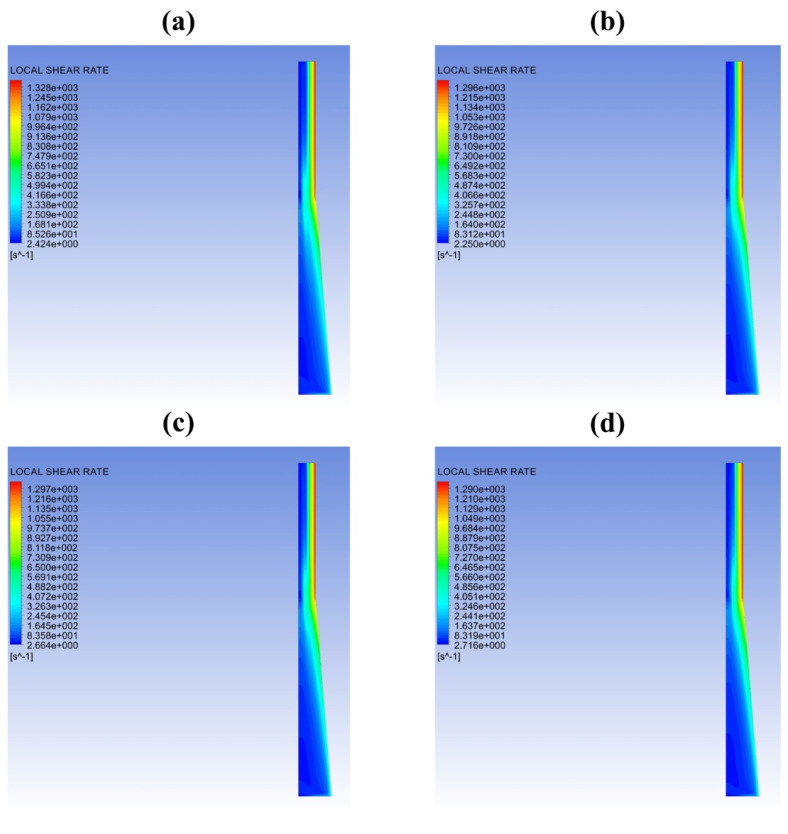
Shear rate contours along the die channel for PET melts at different temperatures with 900 mm^3^/s: (**a**) 271 °C; (**b**) 274 °C; (**c**) 277 °C; (**d**) 280 °C.

**Figure 6:**
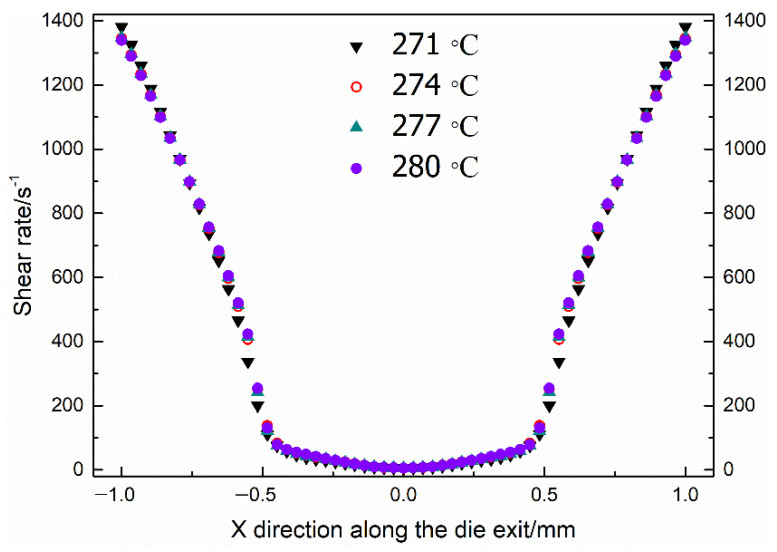
Distributions of the shear rate along the X direction at the die exit.

It is well known that rheological properties significantly affect the bubble growth process. Classic bubble growth theory includes the following rheological properties, such as zero-shear viscosity, characteristic relaxation time, which is based on static batch foaming experiments. It is necessary to acquire the viscosity, the storage modulus and the loss modulus along the die exit in the case of dynamic extrusion situations. However, it is impossible to obtain a specific shear rate distribution with in situ experimental measurements. Figure 5 represents the shear rate contours along the die exit based on CFD simulations. Figure 6 shows the distribution of the shear rate along the X direction at the die exit directly. Owing to the existence of the contraction zone, the shear rate distribution showed two completely different distributions. In the low shear region (LSR), the shear rate changed relatively slowly along the radial direction. On the contrary, the shear rate exhibited a linear increase along the radial direction in the high shear region (HSR). It could be found that the PET melt at 271 °C exhibited the lowest shear rate distribution in the low shear region. Moreover, the shear rate distributions of the PET melt at four different temperatures were almost identical in the high shear region (−1 mm<X<−0.5 mm and 0.5 mm<X<1 mm). Therefore, it was essential to process the rheological data of the low shear region and high shear region, respectively. The distributions of rheological data within the shear rate regions were obtained by substituting the shear rate into the rheological fitting curves, which are illustrated in Figure S2. The relationships between complex viscosity, storage modulus and loss modulus were expressed by [32]:

(8) $$\{\begin{array}{l} \eta =\frac{G\lambda_{c}}{\sqrt{1+\lambda_{c}^{2}{\dot{\gamma }}^{2}}}\\ \begin{array}{c} G^{\prime }=\frac{G\lambda_{c}^{2}{\dot{\gamma }}^{2}}{1+\lambda_{c}^{2}{\dot{\gamma }}^{2}}\\ G^{\prime \prime }=\frac{G\lambda_{c}\dot{\gamma }}{1+\lambda_{c}^{2}{\dot{\gamma }}^{2}} \end{array} \end{array}$$

(9) $$\eta_{a}=\frac{\int_{0}^{S}\eta ds}{S}$$

where η is the shear viscosity, $\dot{\gamma }$ the shear rate, $\lambda_{c}$ the characteristic relaxation time. Using Equations (8) and (9), $\lambda_{c}$ can be calculated from dynamic rheological data with a nonlinear fitting and $\eta_{a}$ is defined as the average shear viscosity at the exit section of the die channel. Table 1 includes the rheological characteristic properties for the low shear region and high shear region. In the low shear region, the characteristic relaxation time of the PET melt at 271 °C was 0.1554. The lower relaxation time indicated that the relaxation and deformation of the polymer melt was faster after the stress was applied under the strong shear. With the increase in the foaming temperature, the polymer segments were excited by external energy to release more chain entanglement. From the perspective of rheological properties, this was reflected as a decrease in the average shear viscosity and characteristic relaxation time.

### 4.3. Determination of Pressure Profile

**Figure 7:**
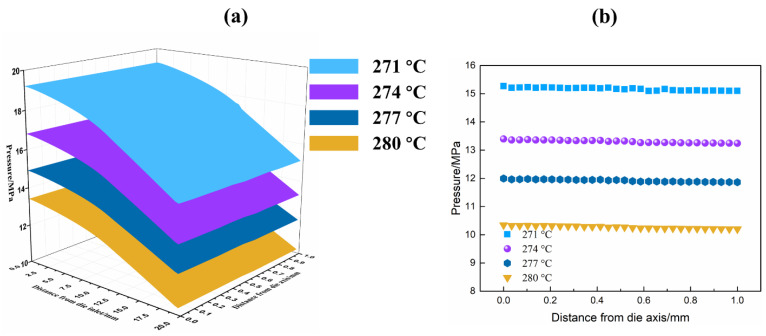
(**a**) Pressure inside the die channel for PET melts and (**b**) pressure along the die exit for PET melt at different temperatures.

Die pressure is an important process parameter for extrusion foaming. For foam growth, cell nucleation and bubble growth have a strong relationship with the foaming pressure. Cell nucleation is affected by the depressurization rate and the depressurization rate which have a strong correlation with the initial die pressure. The melt pressure in the die also determines the solubility of supercritical carbon dioxide in the melt. The solubility of the blowing agent controls the bubble growth. A higher mass transfer driving force can provide more energy to resist the melt stress for the bubble growth process. Therefore, it is essential to obtain the pressure at the die exit for the numerical calculation of the bubble growth. However, the exit of the die is a rather narrow channel with a diameter of 2 mm. It is difficult to measure the actual pressure near the outlet of the die. The inlet of the die can allow the insertion of the pressure sensor to measure the actual melt pressure at this position. Combining boundary conditions and PTT models, the CFD simulation was an effective method to calculate the pressure distribution inside the die channel. Since the cooling effect of the process was stronger, the PET melt with 271 °C established a higher melt pressure at the inlet of the die. Observed from Figure 7, as the temperature decreased, the viscosity of the PET melt had to increase accordingly and the higher melt pressure would appear at the die inlet. Although the die channel was a contraction geometry shape, the energy loss resulting from the polymer flow would be reflected in the pressure drop ultimately. Figure 7 exhibits the pressure variety trends in different directions. It can be seen that the PET melt at 271 °C showed the fastest rate of pressure drops along the die axis. This could be attributed to the high viscoelasticity resulting from the physical entanglements network [33]. These results strongly suggested that excessive viscoelasticity would result in a more serious pressure drop in the channel. Indeed, it may stimulate the nucleation inside the die when the pressure drops exceeded a certain level [34]. This phenomenon probably caused cell deterioration, such as the formation of large cells. In summary, the pressure distributions at the die outlet were calculated through experimental measurement and the CFD simulation, which provided basic data for bubble growth evaluations.

### 4.4. Stress Distribution

**Figure 8:**
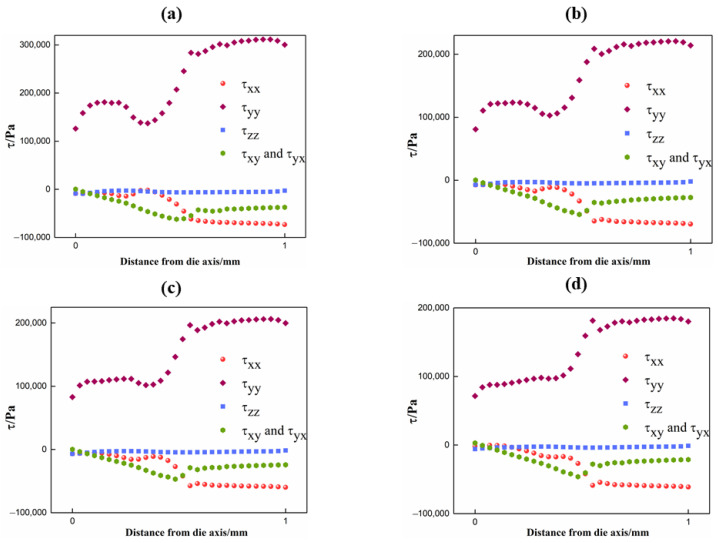
Distribution of stress components along the die exit: (**a**) 271 °C; (**b**) 274 °C; (**c**) 277 °C; (**d**) 280 °C.

When PET fluid flowed into the channel, it experienced large shear and extensional deformations. The calculated fully developed stresses by the PTT model (shear stress components $\tau_{xy}$ and $\tau_{yx}$, normal stress components $\tau_{xx}$, $\tau_{yy}$ and $\tau_{zz}$) at the die exit are shown in Figure 8. In the 2D simulation, the value of other shear stress components, $\tau_{xz}$ and $\tau_{yz}$, were thought to be zero owing to the zero-velocity gradient [27,28,30]. It should be pointed out that the numerical value of $\tau_{yy}$ was almost one order of magnitude larger than $\tau_{xy}$, $\tau_{xx}$ and $\tau_{zz}$. The phenomenon revealed that $\tau_{yy}$ played a key role in the PET melt elasticity. The value of $\tau_{yy}$ decreased with the increase in die temperature, which was consistent with the relationship between the melt elasticity and melt temperature. Moreover, the values of $\tau_{xy}$, $\tau_{xx}$ and $\tau_{zz}$ of the flow conditions were negative. This could be attributed to the restriction effect of the die walls, resulting in the compression flow. In order to simplify calculations, it was reasonable to adopt the average value of the stress components of the die exit cross section to evaluate the bubble growth. The values of the average normal and shear stress are listed in Table S2. However, one of the assumptions in the bubble growth model was that the bubble is spherically symmetric. Hence, it was necessary to convert the stress matrix from the Cartesian coordinate to the spherical coordinate. The specific transformation theory is explained in SI Section S3.

The stress components in the spherical coordinate system are shown in Table 2, which includes the average stress values of the die cross section, the values of the high shear region of the die cross section and the values of the low shear region of the die cross section. It was found that the absolute stress value increased as the temperature decreased whether in the radial direction or in the *θ* direction. This also meant that the melt stress that prevented bubble growth amplified with the decline of die temperature. The precise values of the initial melt stress will further promote the accuracy solution to the bubble growth.

### 4.5. Numerical Simulation of Bubble Growth

#### 4.5.1. Parameter Sensitivity Analysis

In this work, the bubble growth model ignored the plasticization effect of scCO_2_ on the PET melt. It was difficult to accurately measure the high-pressure rheological properties of the polymer/scCO_2_ solution, especially for a high melting point polymer such as PET. Therefore, this hypothesis had been applied in many related works [35,36,37,38,39]. However, the plasticization of scCO_2_ could result in significant changes in surface tension, relaxation time and shear viscosity, which are important parameters in the bubble growth model. To verify the plasticization effect of scCO_2_ on the simulation results, the surface tension, relaxation time and average shear viscosity were selected for a parameter sensitivity analysis. It was necessary to point out that the relevant rheological parameters and thermophysical parameters in the sensitivity analysis were based on the processing conditions and CFD calculation results of the fabrication of PF-271.

**Figure 9:**
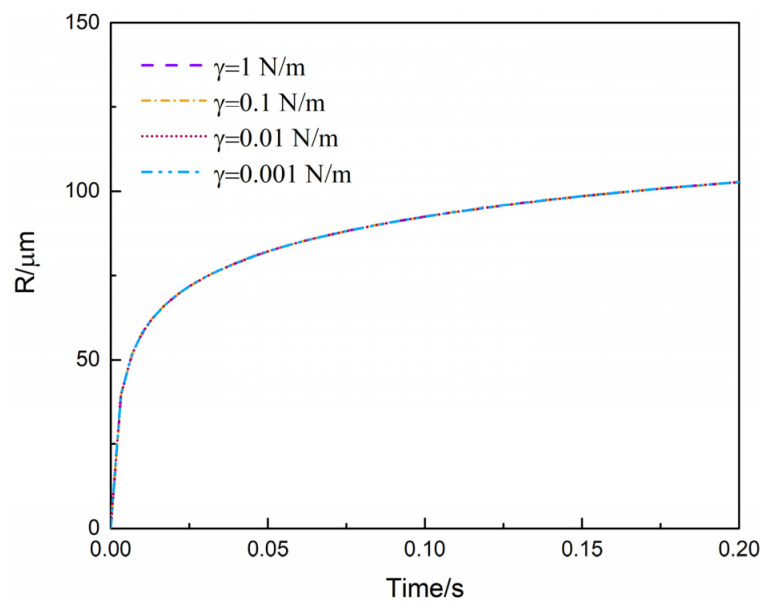
Effect of surface tension (γ) on predicted bubble growth behaviors.

The effect of surface tension on predicted bubble growth behaviors are illustrated in Figure 9. As shown in Figure 9, surface tension had almost no influence on the bubble growth process. Surface tension was a key factor describing the phase separation and cell nucleation according to the Laplace equation and classic nucleation theory [40,41,42,43,44,45]. The surface tension generally accounted for one percent of the viscous resistance. In addition, the effect of static pressure and melt elastic stress was more significant during bubble growth according to Equation (S1). Generally, the surface tensions between the polymer and CO_2_ decreased with the increasing CO_2_ pressure and then leveled off at ultra-high CO_2_ pressures [46].With the surface tension varying from 1N/m to 0.001N/m, the overall bubble growth process was not sensitive to the varieties in surface tension. Hence, the plasticization effect of scCO_2_ on the surface tensions would not reflect on the bubble growth.

**Figure 10:**
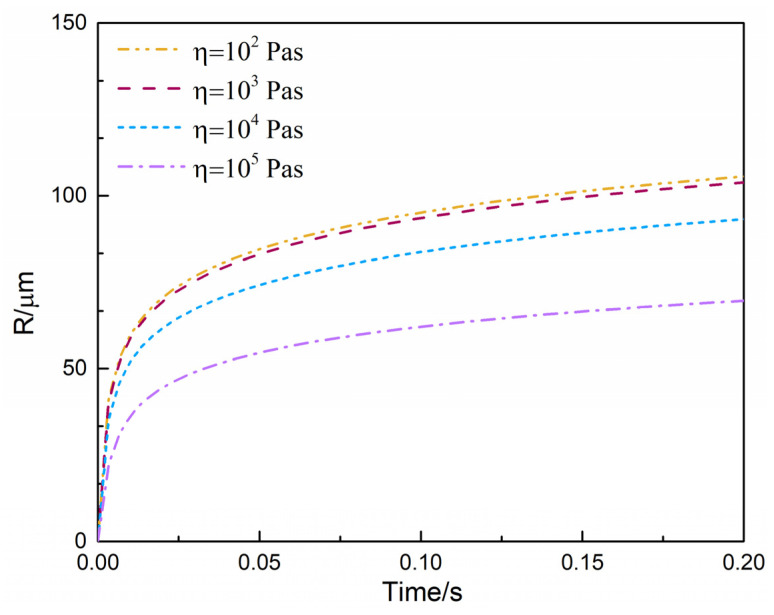
Effect of shear viscosity (*η*) on predicted bubble growth behaviors.

ScCO_2_ effectively expanded the free volume between polymer molecular segments. On the one hand, scCO_2_ could reduce the interaction force between macromolecular segments; on the other hand, scCO_2_ could also promote the mobility of polymer chains. Therefore, the shear viscosity of the PET/CO_2_ solution must be lower than that of the pure PET melt [33,47,48]. It was necessary to investigate the sensitivity of shear viscosity to the bubble growth process. Figure 10 illustrates the effects of shear viscosity on the evaluated bubble growth process where shear viscosity was varied from 10^2^ Pa·s to 10^5^ Pa·s. It can be observed that the high shear viscosity (i.e., $\eta =10^{5}\;\mathrm{Pa}\cdot s$) effectively inhibited the rapid growth of cells. The high shear viscosity meant that cells needed to dissipate more energy during the growth process and accumulate more stress. Therefore, the bubble growth rate was slower compared to other conditions. Moreover, a different shear viscosity resulted in significantly distinct bubble growth processes, which showed that the shear viscosity was highly sensitive to the bubble growth process.

**Figure 11:**
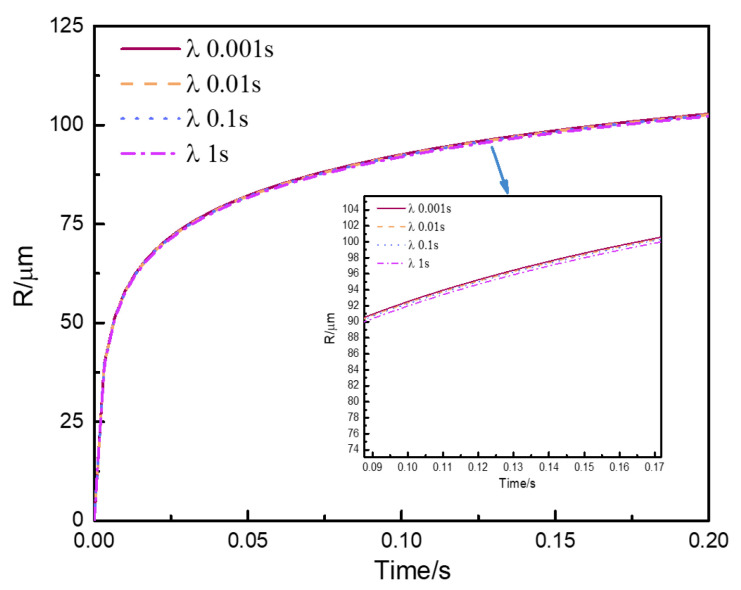
Effect of characteristic relaxation time ($\lambda_{c}$) on predicted bubble growth behaviors.

The effect of characteristic relaxation time on the bubble growth process is shown in Figure 11. Relaxation time was a characterization parameter describing the rate of melt stress relaxation or melt stress accumulation. Figure 11 shows that the rate of bubble growth remained the same in the initial stage of the bubble growth process. In the initial stage, the elongation deformation resulting from the bubble growth process was tiny. It is possible that the melt stress calculated by the constitutive equation was controlled by the initial melt stress. As the elongation deformation resulting from the bubble growth process became larger, the bubble growth curves behaved slightly different. It showed that the influence of the characteristic relaxation time on the melt stress was beginning to appear. Overall, the changes in characteristic relaxation time had no significant effect on the bubble growth, compared to shear viscosity [1,14]. However, the increase in relaxation time may be beneficial to further promote the stability of bubble growth [1,2].

Overall, the sensitivity of shear viscosity to the bubble growth process was more significant than the other two parameters. Therefore, the plasticization effect of scCO_2_ on bubble growth could be attributed to the changes in shear viscosity.

#### 4.5.2. Evaluation of Bubble Growth in scCO_2_ Extrusion Foaming

**Figure 12:**
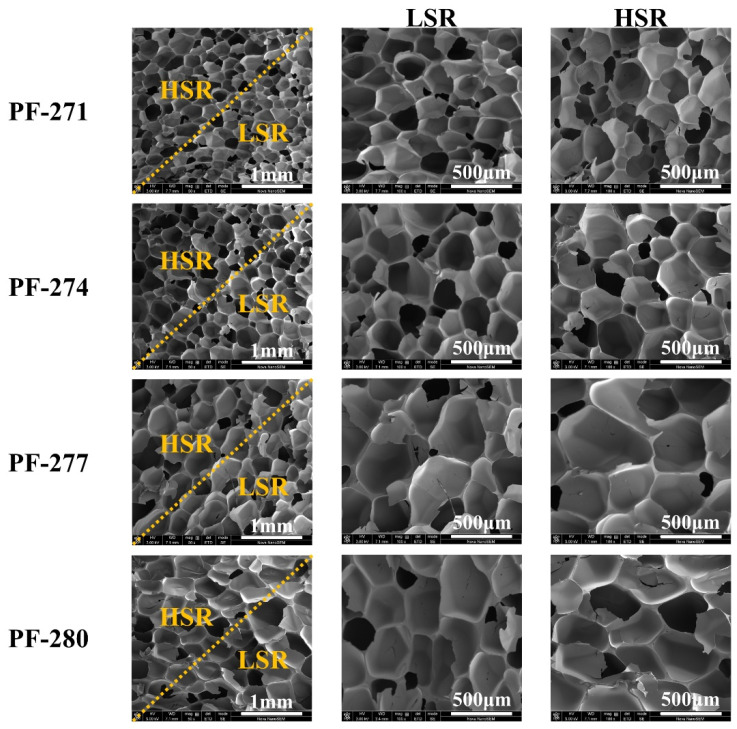
SEM graphs of PET foam morphology.

**Figure 13:**
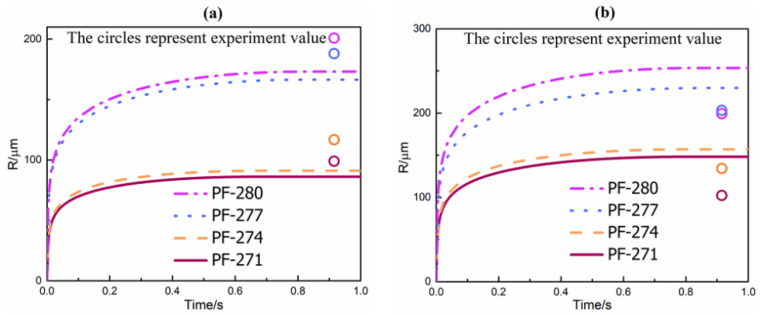
Simulated bubble growth of PET scCO_2_ extrusion foaming: (**a**) low shear region; (**b**) high shear region.

Figure 12 shows the foam morphologies of different PET foam samples in both low shear regions and high shear regions. PF-271 maintained an excellent cell structure; however, PF-280 exhibited significant cell rupture. This could be attributed to the superior bubble growth stability resulting from the longer relaxation time. Figure 13 describes the simulated bubble growth behaviors of PET scCO_2_ extrusion foaming in this work. Specifically, the die cross section was divided into the low shear region and the high shear region to achieve more accurate predictions. Based on the above parameter sensitivity analysis, the plasticization effect of scCO_2_ was ignored in the bubble growth model, so the simulation results may have underestimated the bubble growth. In Figure 13a, the deviations between the simulated results and the experimental values illustrated the effect of plasticization on bubble growth. Although the effect of shear viscosity on bubble growth was consistent, further study about the high-pressure rheological properties of the PET/scCO_2_ system would be required to acquire more accurate predictions for bubble growth. However, it was very challenging and complicated to accomplish the highly accurate quantitative calculation of bubble growth in the extrusion foaming process, because the effect of CO_2_ plasticization on cell growth was dynamic accompanied by changes in the carbon dioxide concentration.

In the high shear region, the bubble growth was less retarded due to a lower shear viscosity and radial melt stress. Therefore, the cells grew faster and the cell size was larger in the high shear region than the low shear region, which was also consistent with the theory [49]. From Figure 13, it can be seen that the simulated results of the high shear region and the low shear region were significantly different. However, it can be easily concluded from Figure 12 that the cell sizes were basically uniform and the experiment values of low shear regions and high shear regions were slightly different. Moreover, Figure 13b shows that the predicted values of the bubble growth model were larger than the experimental values. Actually, the outer surface of the foamed extrudates would undergo a cooling process and the rate of cell solidification would be faster compared to the core of the foamed extrudates [50]. The bubble growth model was based on the isothermal process. It relied on rheological properties and momentum transfer to predict bubble growth behaviors, ignoring the temperature effect. The rapid cooling process in the high shear regions limited the bubble growth and solidified the cells prematurely, which resulted in a smaller cell size than the simulated value. Overall, the simulated values still agreed well with the experiment values, which proved the validity of the model.

**Figure 14:**
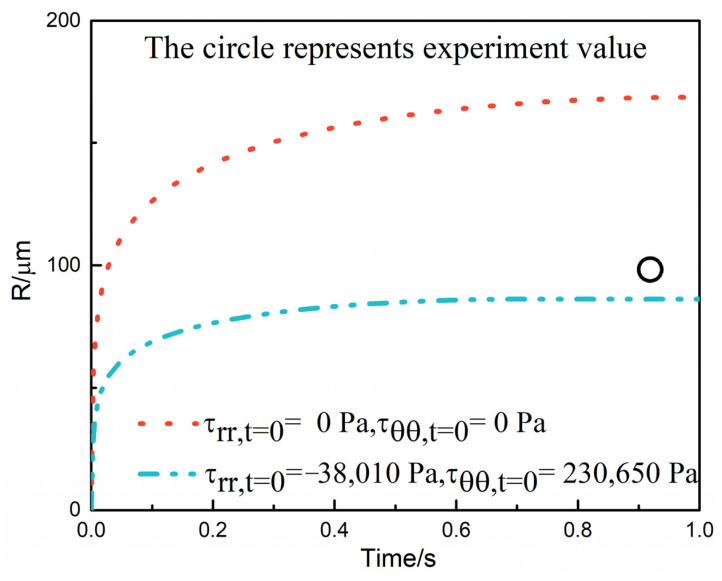
Effect of initial melt stress on bubble growth of PF271.

In many works researched previously, the viscoelastic medium was assumed to be free of initial melt stresses [1,2,14]. With no initial stresses present, the growth was mainly diffusion-controlled. In fact, the surrounding stress domain appeared when the bubbles were nucleated. However, the melt stress components were difficult to acquire through experiment monitoring. Through a CFD simulation, it was possible to calculate the melt stress components [6,51,52,53]. Figure 14 demonstrates the effect of initial melt stresses on the bubble growth. It could be observed from Figure 14 that the hydrodynamic effects were initially dominant and the bubble growth was hindered owing to the melt stresses opposing the bubble expansion. Therefore, the simulated result agreed better with the actual cell sizes of PF-271 assisted with the introduction of initial melt stresses, which illustrated the importance of the initial melt stress components in the bubble growth simulation.

## 5. Conclusions

In this work, we developed a strategy coupled with a CFD calculation and bubble growth model to simulate the bubble growth process in PET scCO_2_ extrusion foaming. PET foams with a varying die melt temperature were fabricated and a cell morphology evolution of PET foams was characterized. The flow condition of the PET melt in the die channel was calculated using the finite element method. The distribution of shear rheological properties, pressure profiles and melt stress components under dynamic situations were obtained and applied to the subsequent bubble growth simulation. The simulation results exhibited that the model predictions agreed well with the experimental results. More importantly, the introduction of the initial melt stress increased the resistance of bubble expansion and predicted the bubble growth process more reasonably compared with the zero value of the initial melt stress. This illustrated that the introduction of the initial melt stress was essential and indispensable based on CFD calculations. The parameter sensitivity of the surface tension, shear viscosity and relaxation time to the bubble growth process was also investigated. Due to the high sensitivity of shear viscosity, the plasticizing effect of scCO_2_ and the cooling effect may have caused some deviation between the simulation and experimental values. Therefore, it is necessary to investigate the change of rheological properties of the PET/scCO_2_ system with a CO_2_ concentration for improving simulation accuracy in future works.

## Figures and Tables

**Table 1:** Calculation of the characteristic relaxation time and average shear viscosity for PET melt at different temperatures.

**Parameter**	271 °C		274 °C		277 °C		280 °C	
	$\eta_{a}$	$\lambda_{c}$	$\eta_{a}$	$\lambda_{c}$	$\eta_{a}$	$\lambda_{c}$	$\eta_{a}$	$\lambda_{c}$
Low shear region	1691	0.1554	1254	0.1006	642	0.0800	569	0.0436
High shear region	181	0.0126	167	0.0018	125	0.0015	94	0.0009

**Table 2:** The values of $\tau_{rr}$ and $\tau_{\theta \theta }$ in different temperatures.

Temperature	Zone	$\tau_{rr}/\mathrm{Pa}$	$\tau_{\theta \theta }/\mathrm{Pa}$
271 °C	Average	−38,010	230,650
	Low shear region	−64,210	297,190
	High shear region	−10,650	164,090
274 °C	Average	−36,100	163,160
	Low shear region	−59,700	212,630
	High shear region	−12,750	117,060
277 °C	Average	−31,560	152,430
	Low shear region	−52,220	199,010
	High shear region	−11,350	108,970
280 °C	Average	−30,930	135,630
	Low shear region	−52,180	178,830
	High shear region	−9682	95,379

## Data Availability

The data presented in this study are available on request from the corresponding author.

## Supplementary Materials

The following are available online at https://www.mdpi.com/article/10.3390/polym13162799/s1, Figure S1: Schematic of a gas bubble and its corresponding polymer/gas solution shell, Figure S2: Rheological properties in the low shear region and high shear region: (a) 271 °C; (b) 274 °C; (c) 277 °C; (d) 280 °C, Table S1: Multi-mode PTT model parameters for PET melts at four different temperatures, Table S2: Average values of normal and shear stress components, Table S3: Thermophysical parameters of the bubble growth simulation.
