# Supplementary material for: Analysis of Bubble Growth in Supercritical CO2 Extrusion Foaming Polyethylene Terephthalate Process Based on Dynamic Flow Simulation
Source: Polymers (Basel). 2021 Aug 20;13(16):2799. doi: 10.3390/polym13162799 (PMC8401983; doi:10.3390/polym13162799)
Supplement: Supplementary file 1 [file polymers-13-02799-s001.zip › polymers-1328823-SI.pdf]

Analysis of bubble growth in supercritical CO_2 PET extrusion foaming process based on dynamic flow simulation

Shun Yao[1], Yichong Chen[1], Yijie Ling[1], Dongdong Hu[1], Zhenhao Xi[1], Ling Zhao[1,2*]

1. Shanghai Key Laboratory of Multiphase Materials Chemical Engineering, East China University of Science and Technology, Shanghai 200237, P. R. China.

2. College of Chemical Engineering, Xinjiang University, Urumqi, 830046, P. R. China

*. Corresponding authors at: Shanghai Key Laboratory of Multiphase Materials Chemical Engineering, East China University of Science and Technology, Shanghai, 200237, China. E-mail address: zhaoling@ecust.edu.cn

Section S1 The mathematical formulations of bubble-growth model

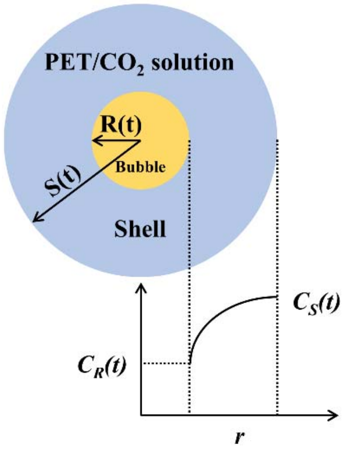

Fig.S1 Schematic of a gas bubble and its corresponding polymer/gas solution shell.

On the basis of the above assumption, the momentum equation is written as:

$$P_b - \frac{2\gamma}{R_b} - P_s + 2\int_{R_b}^{R_s} \frac{\tau_{rr} - \tau_{\theta\theta}}{r} dr = 0 \qquad \text{(S1)}$$

where P_b is the bubble pressure, γ is the surface tension, P_s is the shell pressure. τ_{rr} and $\tau_{\theta\theta}$ are the stress components in the r and θ directions, respectively.

Mass transfer of CO_2 at the interface is generally expressed as below:

$$\frac{d}{dt}\left(\frac{4\pi R_b^3 P_b}{3\Re_g T}\right) = 4\pi R_b^2 D_{gc} \frac{\partial c}{\partial r}|_{r=R} \qquad \text{(S2)}$$

where D_{gc} is the gas coefficient, $\Re_g$ is the gas constant, and c is the gas concentration at the interface. With the following initial and boundary conditions:

$$c|_{r,t=0} = c_0 \qquad \text{(S3)}$$

$$\frac{\partial c}{\partial t}|_{r=S_0,t} = 0 \qquad \text{(S4)}$$

$$c|_{r=R,t} = k_H P_b \qquad \text{(S5)}$$

The mass balance equation for gas in a polymer/CO_2 solution shell is as follows:

$$\frac{\partial c}{\partial t} + v_r \frac{\partial c}{\partial r} = \frac{D_{gc}}{r^2}\frac{\partial}{\partial r}\left(r^2 \frac{\partial c}{\partial r}\right) \qquad \text{(S6)}$$

If the convection term is neglected and the balance equation is shortened by

integrating the assumed polynomial profile, the concentration profile can be simplified as:

$$\frac{d}{dt}(P_b R^3) = \frac{6\left(k_H \Re_g T\right)^2 D_{gc}(P_{b0}-P_b)^2 R^4}{P_b R^3 - P_{b0} R_0^3} \tag{S7}$$

In order to simplify the numerical solution process, the upper-convected Maxwell model is used to calculate the bubble growth process. Using the Lagrangian coordinate transformation, the constitutive equations that characterize the viscoelastic fluid can be reduced to first-order ordinary differential equations [1]:

$$\frac{d\tau_{rr}}{dt} = -4\dot{\varepsilon}\left(\tau_{rr} + \frac{\eta_a}{\lambda_c}\right) - \frac{\tau_{rr}}{\lambda_c} \tag{S8}$$

$$\frac{d\tau_{\theta\theta}}{dt} = 2\dot{\varepsilon}\left(\tau_{\theta\theta} + \frac{\eta_a}{\lambda_c}\right) - \frac{\tau_{\theta\theta}}{\lambda_c} \tag{S9}$$

$$\dot{\varepsilon} = \frac{\dot{R}R^2}{y+R^3} \tag{S10}$$

$$\mathrm{y} = r^3 - R^3 \tag{S11}$$

where λ_c is the characteristic relaxation time of the dynamic PET fluid, $\dot{\varepsilon}$ is the biaxial extension rate, η_a is the average viscosity of the dynamic PET fluid, $\dot{R}$ is the radial velocity at the interface between the gas and the melt. The initial stress conditions $\tau_{rr}|_{y,t=0}$ and $\tau_{\theta\theta}|_{y,t=0}$ are calculated from Polyflow simulation.

Section S2 Rheological properties under dynamic extrusion conditions

Table S1 Multi-mode PTT model parameters for PET melts at four different temperatures.

Mode	271 °C				274 °C				277 °C				280 °C			
	λ_i	η_i	ε_i	ξ_i	λ_i	η_i	ε_i	ξ_i	λ_i	η_i	ε_i	ξ_i	λ_i	η_i	ε_i	ξ_i
1	0.01	752	3.32	0.94	0.01	717	3.40	0.95	0.01	602	3.59	0.97	0.01	553	3.50	1.01
2	0.1	1442	16.85	0.74	0.1	1256	17.32	0.79	0.1	1044	18.21	0.80	0.1	966	18.37	0.83
3	1	10044	0.35	0.024	1	6518	0.37	0.025	1	6749	0.36	0.023	1	5610	0.39	0.026
4	10	118595	0.26	0.21	10	72991	0.26	0.23	10	70320	0.31	0.22	10	49847	0.31	0.27

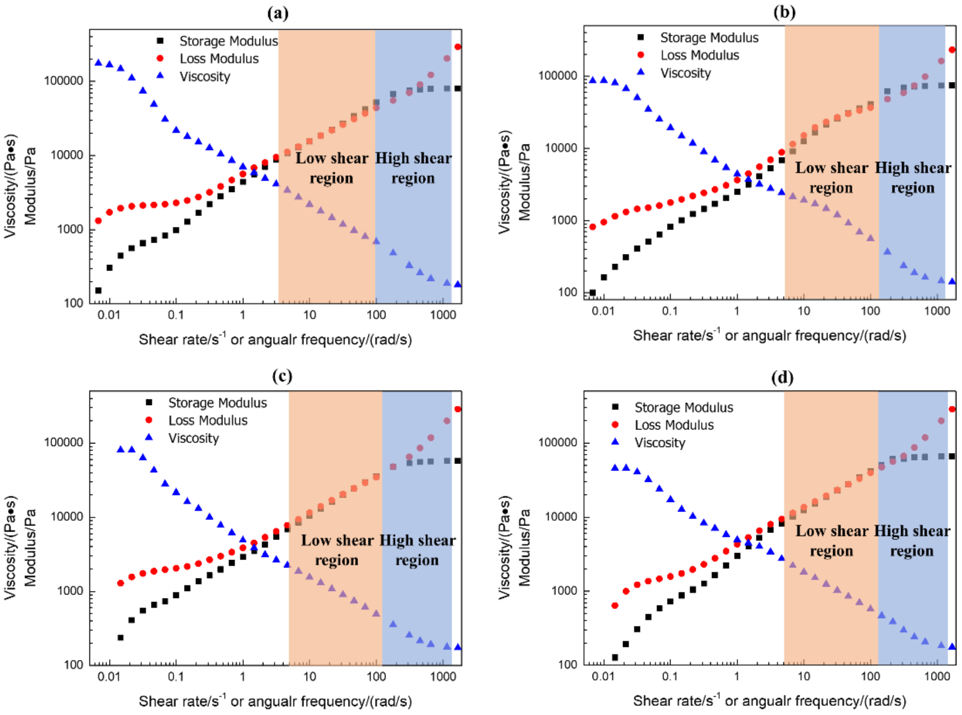

Fig.S2 Rheological properties in the low shear region and high shear region: (a) 271 °C; (b) 274 °C; (c) 277 °C; (d) 280 °C.

Section S3 Calculations of stress components

Table S2 Average values of normal and shear stress components.

Temperature	τ_{xx}/Pa	τ_{yy}/Pa	τ_{zz}/Pa	τ_{xy}/Pa	τ_{yx}/Pa
271 °C	-39127	230775	-5407	-37133	-37133
274 °C	-38093	163271	-4308	-29586	-29586
277 °C	-33066	152520	-3870	-25480	-25480
280 °C	-32753	135699	-3160	-23223	-23223

The stress matrix in Cartesian coordinate σ_c is expressed as:

$$\sigma_c = \begin{bmatrix} \tau_{xx} & \tau_{yx} & \tau_{zx} \\ \tau_{xy} & \tau_{yy} & \tau_{zy} \\ \tau_{xz} & \tau_{yz} & \tau_{zz} \end{bmatrix} \tag{S12}$$

The stress matrix in spherical coordinate σ_s can be obtained by the following equation:

$$\sigma_s = \begin{bmatrix} \tau_{rr} & \tau_{\theta r} & \tau_{\varphi r} \\ \tau_{r\theta} & \tau_{\theta\theta} & \tau_{\varphi\theta} \\ \tau_{r\varphi} & \tau_{\theta\varphi} & \tau_{\varphi\varphi} \end{bmatrix} \tag{S13}$$

$$\sigma_s = \beta_d \sigma_c {\beta_d}^T \tag{S14}$$

$$\beta_d = \begin{bmatrix} \sin\varphi\cos\theta & \sin\varphi\sin\theta & \cos\varphi \\ -\sin\theta & \cos\theta & 0 \\ \cos\varphi\cos\theta & \sin\varphi\sin\theta & -\cos\varphi \end{bmatrix} \tag{S15}$$

where φ and θ are the position variables of the spherical coordinate, which could be solved by the normal stress components.

Section S4 Control parameters of the bubble growth simulation

Accurate thermophysical properties is critical to verify the validity of the mathematical model and the simulation scheme for the bubble-growth behaviors. For the PET/CO_2 system presented in this work, the corresponding parameters are calculated and summarized in **Table S3**, which were based on our previous works adopting magnetic suspension balance measurements and swelling correction.

Table S3 Thermophysical parameters of the bubble growth simulation.

Input data	Value	Reference
Henry constant, k_H(mol/Nm)	8.24×10^{-5}	[4]
Diffusion coefficient, D(m^2/s)	1.00×10^{-9}	[5]
Surface tension, γ(dyne/cm)	$\gamma=39.9-0.0833T-1.6100P+0.0049TP$	[4]
Initial bubble radius (m)	1×10^{-6}	[2]
Initial shell radius (m)	1×10^{-3}	Estimated

Reference

[1] A. Arefmanesh, S.G. Advani, Diffusion-induced growth of a gas bubble in a viscoelastic fluid, Rheologica Acta, 30 (1991) 274-283.

[2] Y. Chen, C. Wan, T. Liu, Z. Chen, Y. Qiao, J. Lu, J. Yan, L. Zhao, M. Esseghir, Evaluation of LLDPE/LDPE blend foamability by in situ rheological measurements and bubble growth simulations, Chemical Engineering Science, 192 (2018) 488-498.

[3] Y. Li, Z. Yao, Z.-h. Chen, K. Cao, S.-l. Qiu, F.-j. Zhu, C. Zeng, Z.-m. Huang, Numerical simulation of polypropylene foaming process assisted by carbon dioxide: Bubble growth dynamics and stability, Chemical Engineering Science, 66 (2011) 3656-3665.

[4] Z. Hua, S. Sun, Z. Xi, T. Liu, L. Zhao, Solubility of CO_2 in molten poly(ethylene terephthalate), CIESC Journal, 64 (2013) 1513-1519.

[5] S. Areerat, E. Funami, Y. Hayata, D. Nakagawa, M. Ohshima, Measurement and prediction of diffusion coefficients of supercritical CO2 in molten polymers, Polymer Engineering & Science, 44 (2004) 1915-1924.
